# Presenting signs and clinical outcome in dogs with metaphyseal osteopathy: 39 cases (2009–2018)

**DOI:** 10.1111/jsap.13554

**Published:** 2022-09-19

**Authors:** A. L. Robertson, V. L. Black, E. Villedieu, K. E. Clarke, I. Faux, A. Major, S. Adamantos

**Affiliations:** ^1^ Bristol Veterinary School, University of Bristol Bristol BS8 1TH UK; ^2^ The Ralph Veterinary Referral Centre, The Ralph Veterinary Referral Centre Marlow SL7 1YG UK; ^3^ Willows Veterinary Centre and Referral Service, Willows Veterinary Centre and Referral Service Shirley B90 4NH UK; ^4^ Davies Veterinary Specialists, Davies Veterinary Specialists Shillington SG5 3HR UK; ^5^ Royal (Dick) School of Veterinary Studies, University of Edinburgh Edinburgh EH25 9RG UK; ^6^ Langford Vets Small Animal Referral Hospital, University of Bristol Langford BS40 5DU UK

## Abstract

**Objective:**

To describe the presenting signs, concurrent conditions, treatment and outcome of dogs with metaphyseal osteopathy.

**Materials and Methods:**

Multi‐centre retrospective review of medical records from January 2009 to September 2018 at four referral centres to identify dogs with a radiographic diagnosis of metaphyseal osteopathy.

**Results:**

Thirty‐nine dogs were identified. The median age at onset was 14 weeks old (range, 8 to 32 weeks old). There was a higher proportion of male dogs (29 of 39 male entire, nine of 39 female entire, one of 39 female neutered and no male neutered dogs). Where information was available, median time from the most recent vaccination was 20 days (range, 2 to 144 days). The most commonly recorded clinical signs were pyrexia (34 of 39), lethargy (32 of 39), pain (30 of 39), and being non‐ambulatory (17 of 39). Thirty‐five dogs required hospitalisation for analgesia and supportive care, 19 of 39 were discharged on prednisolone (median dose 2.0 mg/kg/day; range, 0.9 to 2.6 mg/kg/day), 18 of 39 were discharged on non‐steroidal anti‐inflammatories, two of 39 did not receive NSAIDs or prednisolone at any time point. The median duration of hospitalisation for those admitted was 5 days (range, 1 to 21 days). Where follow‐up was available, relapse occurred in eight of 25 cases before reaching skeletal maturity. At the time of metaphyseal osteopathy diagnosis, five of 39 cases had concurrent conditions. Where follow‐up was available, four of 25 developed future immune‐mediated conditions.

**Clinical Significance:**

Metaphyseal osteopathy should be considered in non‐ambulatory painful young dogs. Some dogs developed future immune‐mediated conditions, which may support the hypothesis that metaphyseal osteopathy is an autoinflammatory bone disorder. Further studies with a larger cohort are required to determine the clinical significance of this.

## INTRODUCTION

Metaphyseal osteopathy is a rare bone disorder affecting young growing dogs between 2 and 8 months old (Grøndalen 1976, Özer *et al*. 2004, Safra *et al*. 2013). Commonly, long bones such as the radius, ulna and tibia are affected (Woodard 1982, Abeles *et al*. 1999). Other sites such as the humerus, ribs, scapula, femur, mandible and optic foramina have also been reported (Watson *et al*. 1973, Woodard 1982, Franklin *et al*. 2008). Large breeds are overrepresented, such as great Danes, Irish setters, Chesapeake Bay retrievers, Weimaraners and German shepherd dogs (Munjar *et al*. 1998, LaFond *et al*. 2002).

Common clinical signs include pyrexia, ostealgia and lethargy (Grøndalen 1976, Abeles *et al*. 1999, Safra *et al*. 2013). A radiolucent line in the metaphysis parallel to the epiphyseal plate is supportive of metaphyseal osteopathy (Watson *et al*. 1973, Grøndalen 1976, Demko & McLaughlin 2005). Histopathology of affected bones shows symmetrical, suppurative sterile osteomyelitis, periosteal proliferation and increase in osteoclast numbers (Mee *et al*. 1993, Joiner & Montgomery 2011).

The aetiology of metaphyseal osteopathy remains unknown. Previous theories of vitamin C deficiency (Watson *et al*. 1973) and overnutrition (Hedhammar *et al*. 1974, Teare *et al*. 1979) have since been disproven (Grøndalen 1976, Woodard 1982). A link to vaccination and canine distemper virus has also been suggested; however, findings have failed to be reproduced (Mee *et al*. 1993, 1995a,b, Munjar *et al*. 1998). In light of this, alternate theories have been proposed. One theory is that there may be a genetic predisposition to metaphyseal osteopathy in certain breeds. Entire litters and closely related Weimaraners have been affected, supportive of heritability in this breed (Woodard 1982, Abeles *et al*. 1999, Harrus *et al*. 2002, Safra *et al*. 2013, 2016). This has also been reported in Australian Kelpie littermates (Greenwell *et al*. 2014). Recently, it has been proposed that metaphyseal osteopathy is an immune‐mediated disease as affected dogs have a cytokine profile similar to that of children with autoinflammatory bone conditions (Safra *et al*. 2016).

Optimal treatment has not been determined. Treatment is usually supportive with analgesia including either non‐steroidal anti‐inflammatory drugs (NSAIDs) or corticosteroids (Grøndalen 1976, Abeles *et al*. 1999, Demko & McLaughlin 2005). In a recent study, Weimaraners treated with prednisone had more rapid resolution of clinical signs and shorter length of hospitalisation compared with those treated with NSAIDs (Safra *et al*. 2013). Outcome is generally good; however, relapse episodes can be seen until growth plate closure (Safra *et al*. 2016).

The aim of this study was to describe the presenting signs, concurrent conditions, treatment and outcome in dogs with metaphyseal osteopathy.

## MATERIALS AND METHODS

### Study design and inclusion criteria

A multi‐centre retrospective case series of 39 dogs with a radiographic diagnosis of metaphyseal osteopathy, presenting between 2009 and 2018.

### Medical record search

Medical databases were searched in October 2018 by one operator at each of the four participating four referral centres. A search was performed to identify dogs with a final diagnosis of metaphyseal osteopathy presenting between January 2009 and September 2018.

### Diagnostics performed

Diagnosis was based on clinical signs and radiographic evidence of radiolucency parallel to the metaphysis on radiographs or CT images (Selman & Towle Millard 2022). For cases in which a contemporaneous imaging report was not accessible, radiographs, where available, were retrieved and reviewed by a specialist in diagnostic imaging.

### Data extracted

Data describing the radiographic changes and bones affected were recorded. The following information was collected from the medical records: signalment, vaccination status, type of vaccine administered and time between last vaccination and onset of clinical signs, clinical signs, history and physical examination findings, treatment and length of hospitalisation. Pyrexia was defined as an increased body temperature (≥39.2°C). Recent vaccination was defined as dogs vaccinated within 30 days before presentation at the referral centre.

The medical records at the diagnosing hospital were reviewed for evidence of relapse and referring veterinary practices were contacted for follow‐up information. Follow‐up information included any relapse episodes or development of any future conditions. Relapse was defined as a recurrence of clinical signs before reaching skeletal maturity. Future conditions were defined as new conditions which developed after the dog reached skeletal maturity and were included if the information was available about how the diagnosis was reached.

Data were presented descriptively. For normally distributed variables, descriptive statistics are presented as the mean ±sd. Non‐normally distributed variables were presented as the median and range.

## RESULTS

### Signalment

Thirty‐nine dogs were identified. Median age at presentation was 14 weeks (range, 8 to 32 weeks). The most common breeds were Border collie (six of 39; 15.3%), Weimaraner (five of 39; 12.8%) and Labrador retriever (five of 39; 12.8%). There was a higher proportion of male dogs; 29 of 39 (74.3%) dogs were male entire, nine of 39 (23.0%) were female entire, one of 39 (2.6%) was female neutered, no dogs were male neutered.

### Vaccination

History regarding vaccination status was available for 37 of 39 (94.9%) dogs; 36 of 37 (97.3%) were vaccinated. The median time from the most recent vaccination to the onset of clinical signs was 20 days (range, 2 to 144 days). Twenty‐four (66.7%) vaccinated dogs had been recently vaccinated.

Of the dogs vaccinated, the type of vaccination administered was available in 27 of 37 (73.0%) dogs, protocols are described in Table S1. All dogs received live attenuated vaccines. The most common vaccination protocol was Nobivac^Ⓡ^ DHPPi (for *distemper*, canine *adenovirus type 2*, *canine parvovirus and canine parainfluenza*) in combination with the Nobivac^Ⓡ^ inactivated L2 vaccine (for *Leptospirosis interrogans serogroup canicola and icterohaemorrhagiae*) (11 of 27; 40.7%).

### Clinical signs

The most common clinical sign in dogs presenting to their primary care practice was pyrexia (34 of 39; 87.2%), leading to referral to one of the four referral centres. Pyrexic dogs had a median rectal temperature of 40.0°C (range, 39.2 to 41.2°C). Most dogs were lethargic (32 of 39; 82.1%) and painful (30 of 39; 76.9%). In the painful dogs, localisation of pain was reported in 22 of 30 (73.3%). This was localised to joint pain (13 of 22; 59.0%), long bone and joint pain (five of 22; 22.7%) or a pain response on long bone palpation (four of 22; 18.2%). Seventeen (43.6%) were non‐ambulatory on presentation. Eight had a reduction in appetite [hyporexia (four of eight; 50.0%), anorexia (four of eight; 50.0%)]. Other clinical signs were reluctance to walk (six of 39; 15.4%), vomiting (four of 39; 10.3%), jaw pain (four of 39; 10.3%), neck pain (three of 39; 7.7%), nasal discharge (two of 39; 5.1%), cough (two of 39; 5.1%) and diarrhoea (two of 39; 5.1%).

At the time of diagnosis of metaphyseal osteopathy, five (12.8%) dogs had concurrent conditions (Table 1). Three dogs (7.7%) were diagnosed with inflammatory conditions, suspected to be immune‐mediated. These were juvenile cellulitis (one of three; 33.3%), vasculitis (one of three; 33.3%) and rhinitis (one of three; 33.3%). Two dogs (5.1%) had conjunctivitis at the time of diagnosis. One dog (2.6%) was diagnosed with craniomandibular osteopathy. An angular limb deformity was present in one dog (2.6%) at the time of presentation; this was assumed to have developed as a sequala of metaphyseal osteopathy. Three dogs (7.7%) had synoviocentesis performed; synovial fluid analysis revealed neutrophilic joint inflammation in all three (100%) dogs.

**Table 1:** Description of dogs with concurrent conditions at time of metaphyseal osteopathy diagnosis and long‐term outcome

Dog	Concurrent condition(s)	How diagnosis of concurrent condition was made	Did the dog have relapse episode(s)	Did the dog develop future condition(s)
1	Juvenile cellulitis Conjunctivitis	Cytology and bacterial culture of pustular dermatitis Ophthalmic examination	No	No
2	Vasculitis Conjunctivitis Neutrophilic joint effusion Polyradiculoneuritis Protein‐losing nephropathy	Clinical examination Ophthalmic examination Cytology of synovial samples from both stifles, hocks, carpi Nerve and muscle biopsies, electrodiagnostics urinalysis, abdominal ultrasound, renal fine needle aspirates	No	No
3	Craniomandibular osteopathy	CT scan	No	Yes – angular limb deformity
4	Rhinitis	CT scan, rhinoscopy and nasal swab for culture (negative for bacterial and fungal infections)	Unknown (no follow‐up)	Unknown (no follow‐up)
5	Angular limb deformity (unilateral carpal valgus)	Radiography	Unknown (no follow‐up)	Unknown (no follow‐up)

### Radiographic changes

Thirty‐five dogs (89.7%) had metaphyseal osteopathy diagnosed on radiography, the other four (10.3%) dogs were diagnosed by CT. The most commonly affected anatomical regions were distal radius (24 of 39; 61.5%), distal ulna (24 of 39; 61.5%), distal tibia (12 of 39; 30.7%) and proximal tibia (eight of 39; 20.5%) (Table 2). All dogs had radiolucent lesions parallel to the metaphysis. Other frequently reported lesions were periosteal reaction (six of 39; 15.4%), soft tissue swelling (six of 39; 15.4%), sclerotic bone (six of 39; 15.4%) and new bone formation (five of 39; 12.8%). Five dogs (12.8%) had radiographic changes considered concurrent to the diagnosis of metaphyseal osteopathy. These were angular limb deformity (one of five; 20.0%), craniomandibular osteopathy (one of five; 20.0%), popliteal lymph node enlargement (one of five; 20.0%), reduction in size of the medial trochlear ridge of the tali (one of five; 20.0%) and turbinate destruction of the right nostril (one of five; 20.0%) (Table 3). Changes found on CT scan performed in four of 39 (10.3%) dogs are described in more detail in Table 4. One dog (25.0%) had lymphadenopathy reported, otherwise radiographic changes reported were all musculoskeletal.

**Table 2:** Anatomical region that radiographic metaphyseal changes were present and frequency of this localisation

Bone affected	Number of dogs
Distal radius	24
Distal ulna	24
Distal tibia	12
Proximal tibia	8
Distal fibula	5
Proximal humerus	5
Proximal radius	4
Distal femur	4
Proximal femur	3
Proximal ulna	3
Distal humerus	3
Metacarpals	3
Metatarsals	3
Mandibular condyles	1
Rib	1
Vertebra (caudal C5)	1

**Table 3:** Radiographic changes reported and frequency of dogs in which these were present

Radiographic changes present	Number of dogs
Radiolucency parallel to metaphysis	39
Periosteal reaction	6
Sclerotic bone	6
Soft tissue swelling	6
New bone formation	5
Metaphyseal widening (“flare”)	3
Carpal valgus	1
Craniomandibular osteopathy	1
Popliteal lymph node enlargement	1
Turbinate destruction of right nostril	1
Reduced size of medial trochlear ridge of tali	1

**Table 4:** Description of changes found on CT scan

Dog	Areas scanned	CT radiographic changes
1	Head, thorax, abdomen, proximal forelimbs	Hypoattenuating band and hypoattenuating lesions distal to the proximal humeral metaphyses Radiolucent lesion in the distal aspect of the right eighth rib Lymphadenopathy of cranial mediastinal, sternal and mesenteric lymph nodes
2	Head, forelimbs	Bilateral radius, ulna and humeral metaphyses were mottled with irregular regions of osteolysis. Mandibular condyles had moderate irregular hypoattenuation of subchondral bone and multi‐focal disruption of cortical bone
3	Head, forelimbs	Bilateral radius and ulna had a radiolucent band running parallel to the distal physis with surrounding sclerosis and moderate smooth periosteal reaction Thinning and reduced distinction of central part of ulnar growth plates
4	Head, forelimbs	Bilateral antibrachial bones and humerus had osteopenia in the metaphyses. Defined lesions running in transverse orientation to long bone axis (trummerfield zones). Slight periosteal reaction associated with the lesions (Pelkan spurs)

### Length of hospitalisation

Thirty‐five dogs (89.7%) were hospitalised for treatment. Median length of hospitalisation was 5 days (range, 1 to 21 days). The dogs that were not hospitalised were all prescribed NSAIDs [carprofen (two of four; 50.0%), firocoxib (one of four; 25.0%), meloxicam (one of four; 25.0%)]; the dog that was prescribed meloxicam also received methadone at the time of presentation. All dogs survived to discharge.

### Treatment

Most dogs (37 of 39; 94.9%) received either prednisolone (19 of 39; 47.5%) or NSAIDs (18 of 39; 46.1%). Two dogs (5.2%) were not prescribed prednisolone or NSAIDs at any time during hospitalisation or upon discharge from the hospital. As inpatients, both dogs received opioid analgesia (buprenorphine or methadone). One dog was discharged on paracetamol, the other was discharged with no medication. Supportive care (as described by Selman & Towle Millard 2022) was prescribed to dogs at the clinician's discretion. This constituted analgesia with opioid medication (28 of 39; 71.8%); the most commonly prescribed opioids were tramadol (13 of 28; 46.4%), methadone (eight of 13; 28.6%), buprenorphine (eight of 28; 28.6%). Other analgesics were given whilst hospitalised; these were paracetamol (14 of 39; 35.9%), ketamine (four of 39; 10.3%), lidocaine (two of 39; 5.1%) and gabapentin (one of 39; 2.6%). Dogs were started on intravenous fluid therapy if not eating. Other medication given whilst in hospital included: maropitant (three of 39; 7.7%), omeprazole (two of 39; 5.1%), ranitidine (two of 39; 5.1%). Seven (17.9%) dogs received intravenous antibiotics whilst hospitalised. One dog (one of 39; 2.6%) was prescribed each of the following: metoclopramide, mirtazapine, probiotics, pentoxifylline and benazepril. One dog (2.6%) had a urinary catheter placed for bladder management due to it being non‐ambulatory.

Eighteen dogs (46.2%) were discharged on NSAIDs [meloxicam (n=11), carprofen (n=6) and firocoxib (n=1)] and did not receive prednisolone at any point during hospitalisation. Eleven dogs (28.1%) failed to respond to NSAIDs [meloxicam (nine of 11; 81.8%), meloxicam and robenacoxib (one of 11; 9.1%), carprofen (one of 11; 9.1%)]. These non‐responders were transitioned to receive prednisolone whilst hospitalised; they were included within the prednisolone group data. Of the nineteen (47.5%) dogs that received prednisolone the dose was recorded in 17 dogs; the median dose was 2.0 mg/kg/day (range, 0.9 to 2.6 mg/kg/day).

Length of treatment course advised by the clinician following discharge was available for the 25 dogs that had follow‐up information. In these dogs, 14 of 25 (56.0%) received prednisolone; median length of treatment advised was 20 days (range, 13 to 210 days). Nine (36.0%) dogs were discharged on NSAIDS [meloxicam (four of nine; 44.4%), carprofen (three of nine; 33.3%), firocoxib (one of nine; 11.1%), robanocoxib (one of nine; 11.1%)]; median length of treatment advised was 13 days (range, 5 to 60 days). Two dogs (8.0%) were discharged without prednisolone or NSAIDs; of these, one was prescribed paracetamol, the other was discharged without any medication. Dogs were also prescribed additional treatment following discharge. Further analgesia was given: paracetamol (seven of 39; 17.9%) and tramadol (six of 39; 15.4%). Antacids were prescribed in two (5.1%) dogs: ranitidine (one of 39; 2.6%), omeprazole (one of 39; 2.6%). Two dogs (5.1%) were prescribed antibiotics; one was prescribed cephalexin (7 days), metronidazole (7 days) and marbofloxacin (7 days), the other dog was prescribed cephalexin (2 weeks). Other medications were probiotic paste (one of 39; 2.6%), benazepril (one of 39; 2.6%) and fuscidic acid (one of 39; 2.6%).

### Outcome

Follow‐up information was available for 25 dogs (64.1%); median length of follow‐up after diagnosis was 3.2 years (range, 10 days to 9 years). Relapse occurred in eight of 25 (32.0%) dogs; three of eight (37.5%) suffered from multiple relapse episodes. Median time from diagnosis to relapse was 50 days (range, 7 to 212 days).

Of the dogs that relapsed, management of the initial metaphyseal osteopathy episode was as follows: five of eight (62.5%) were discharged on a tapering course of prednisolone (three of five (60.0%) of these had initially failed to respond to a course of NSAIDs), the other three of eight (37.5%) were discharged on non‐steroidal anti‐inflammatory medication [meloxicam (two of eight; 25.0%), carprofen (one of eight; 12.5%)]. At the time of relapse, four of eight dogs (50%) were on a course of NSAIDs or prednisolone. Of the dogs that relapsed, three of eight (37.5%) went on to develop future conditions (Table 5.).

**Table 5:** Description of relapse episode(s), treatment protocols at time of discharge from hospital after the initial episode of metaphyseal osteopathy and outcome for cases in which relapse occurred (n=8)

Dog	Number of relapse episodes	Length of time from diagnosis to relapse	Treatment after initial episode	Length of treatment	On treatment at time of relapse?	Future conditions
1	2	14 days/50 days	Meloxicam	Unknown	Y meloxicam at both	Sterile panniculitis, meningomyelitis and myositis, vestibular disease
2	1	81 days	Prednisolone	9.5 weeks	N	Suspected septic foci leading to endocarditis, neurological signs, multi‐systemic neutrophilic inflammation, hypercalcaemia
3	1	49 days	Prednisolone	23 weeks	Y	None
4	2	18 days/150 days	Prednisolone	2 weeks initial	N at first Y prednisolone at second	IMPA, right‐sided hemiparesis
5	2	60 days/212 days	Meloxicam	Unknown	Y N at second	None
6	1	150 days	Prednisolone	Unknown	N	None
7	1	7 days	Carprofen	4 days	N	Unknown
8	1	15 days	Prednisolone	Unknown	Y	None

Where follow‐up information was available, five dogs (20.0%) developed future conditions (Table 6). One dog acquired an angular limb deformity (ALD) 4 weeks after the diagnosis of metaphyseal osteopathy. This dog had been diagnosed with craniomandibular osteopathy at the time of metaphyseal osteopathy diagnosis; the ALD was not present during the initial investigations. The other dogs (four of 25; 16.0%) all developed a future immune‐mediated disease: panniculitis (one of four; 25.0%), menigiomyelitis and myositis (one of four; 25.0%), vasculitis (one of four; 25.0%), IMPA (one of four; 25.0%) and lymphadenitis (one of four; 25.0%). IMPA was diagnosed by synovial fluid analysis and radiographic evidence of resolution of the pathology associated with previously diagnosed metaphyseal osteopathy. Of the dogs that developed future conditions, three of five (60.0%) had relapses of metaphyseal osteopathy, with two of three (66.7%) suffering two relapse episodes.

**Table 6:** Description of the future conditions developed in dogs, including how diagnosis was reached and if the dog suffered from previous metaphyseal osteopathy (MO) relapse episodes

Dog	Future condition(s)	How long after MO diagnosis the condition developed	How diagnosis of future condition was made	Did the dog have concurrent conditions at the time of MO diagnosis?	Did the dog have relapse episode(s)?
1	Vestibular disease Sterile panniculitis (affecting mammary tissue) Meningiomyelitis and myositis	1 year 3 months 1 year 5 months 3 years 7 months	Clinical signs, ear examination revealed otitis externa (due to *Malassezia* species), head CT unremarkable Ultrasound of mammary tissue, cytology of fine needle aspirates, histopathology of trucut biopsies and negative tissue culture Magnetic resonance imaging (MRI), cerebrospinal fluid (CSF) cytology and negative culture, histopathology of muscle, negative tissue culture on muscle	No	Yes – 2 episodes
2	Vasculitis Deep pyoderma (*Escherichia coli*)	15 days	Histopathology of skin biopsies Bacterial and fungal culture of skin biopsies	No	No
3	Immune‐mediated polyarthritis (IMPA) right‐sided paresis	11 months 10 days after IMPA diagnosis	Radiography of limbs unremarkable (showed resolution of previous pathology from metaphyseal osteopathy) Synovial fluid analysis Clinical signs	No	Yes – 2 episodes
4	Angular limb deformity (bilateral carpal valgus)	4 weeks	Radiographs	Yes – craniomandibular osteopathy	No
5	Multi‐systemic neutrophilic inflammation (respiratory tract and bicavitary effusions) Lymphadenitis Oesophagitis Suspected septic foci leading to endocarditis, neurological signs	18 months 18 months 18 months 4 years 9 months	Abdominal ultrasound, thoracic radiography, CT scan. Abdominal and pleural effusion analysis (cytology and negative bacterial culture) Fine needle aspirate of retropharyngeal lymph node (cytology and negative bacterial culture) Endoscopy Echocardiography, neurological clinical signs		Yes – 1 episode

## DISCUSSION

This study describes the presenting signs, concurrent conditions, treatment and outcome in dogs with metaphyseal osteopathy. There were a high proportion of males in our population. Previous studies containing various breeds found males were 2.3 times more likely to develop metaphyseal osteopathy compared with females (Munjar *et al*. 1998, Özer *et al*. 2004); this finding was not identified in Weimaraners (Crumlish *et al*. 2006, Safra *et al*. 2013).

Treatment for dogs with metaphyseal osteopathy in this cohort of dogs included analgesia and anti‐inflammatory medication with either NSAIDs or prednisolone. In our group, treatment protocols were based upon clinician preference and subjective assessment of response to treatment. Supportive treatment typically consists of intravenous fluid therapy, analgesia, nutritional support, gastrointestinal protectants and nursing care (Selman & Towle Millard 2022). Many dogs received NSAIDs as first‐line therapy. Several different NSAIDs were used with a failed response subjectively evaluated by the attending clinician. It is therefore difficult to assess if dogs may have responded to NSAIDs had a different class been given or a standard protocol been followed. In dogs perceived to have failed NSAID treatment the NSAID was discontinued and prednisolone was prescribed. Prednisolone was administered to most dogs at an immunosuppressive dose (median 2.0 mg/kg/day; range, 0.9 to 2.6 mg/kg/day). In a case series of Weimaraners with metaphyseal osteopathy, there was a quicker resolution of clinical signs when treated with prednisone compared with those treated with NSAIDs (Safra *et al*. 2013). Prednisone dosage (0.75 to 1.5 mg/kg twice daily) was similar to that of prednisolone used in our population (0.9 to 2.6 mg/kg/day), however, optimal dosage remains unknown.

It has been hypothesised that metaphyseal osteopathy is induced by vaccination (Mee *et al*. 1993, 1995a,b). Within our study recent vaccination was defined as within 30 days before presentation, as reported previously (Duval & Giger 1996, Safra *et al*. 2013). Using this definition, 24 of 36 (66.7%) of dogs had been recently vaccinated. Previous authors have suggested that development of metaphyseal osteopathy is triggered by recent vaccination and that an alternative vaccination protocol, with a killed vaccine, should be considered for Weimaraners. This was proposed due to the suspicion of immunodeficiency in this breed (Day *et al*. 1997, Abeles *et al*. 1999, Harrus *et al*. 2002). However, the hypothesis that metaphyseal osteopathy is linked to vaccination is not supported by other study findings (Munjar *et al*. 1998, Crumlish *et al*. 2006).

It has been suggested that vaccination can act as a trigger for the development of immune‐mediated conditions in people, such as immune‐mediated thrombocytopaenia (ITP) (Black *et al*. 2003). Currently, it is unclear whether vaccination in dogs can act as a trigger for autoimmune diseases such as ITP (Huang *et al*. 2012) and immune‐mediated haemolytic anaemia (Garden *et al*. 2019). As metaphyseal osteopathy is a juvenile disease and a primary course of vaccination is commonly administered to young dogs (Day *et al*. 2016, The People's Dispensary for Sick Animals 2018), it is likely that dogs with metaphyseal osteopathy have been recently vaccinated. It is therefore not possible to prove causation between recent vaccination and the development of metaphyseal osteopathy.

Clinical signs most commonly reported within our population were pyrexia (34 of 39; 87.2%), lethargy (32 of 39; 82.0%) and pain (30 of 39; 76.9%); this is in line with the presentation of dogs in prior studies. Our dogs and those in previous study populations also commonly suffered from gastrointestinal signs (Abeles *et al*. 1999, Safra *et al*. 2013). Affected dogs can also show concurrent jaw pain and nasal discharge (Safra *et al*. 2013). Dermatological changes, such as juvenile cellulitis, were present within our study and in previous groups of puppies (Wentzell 2011, Safra *et al*. 2013).

Radiographic lesion distribution and changes were described within our study. Most dogs had metaphyseal osteopathy diagnosed on radiographs, the others were diagnosed on CT scan. Due to the retrospective nature of the study, it was not possible to determine the clinician's reason for choosing a different imaging modality for these dogs. One dog had lymphadenopathy detected; the remainder had musculoskeletal changes which would be detected on radiographs. In this population of dogs, there appeared to be limited benefit of CT scan compared with radiography for diagnosing metaphyseal osteopathy and concurrent conditions. Lesions commonly were found in long bones and less frequently in other areas (rib, vertebra, mandible, metacarpals, metatarsals); this was in keeping with previous reports (Abeles *et al*. 1999, Franklin *et al*. 2008, Joiner & Montgomery 2011, Greenwell *et al*. 2014). The lesions associated with metaphyseal osteopathy are thought to represent a spectrum of disease, with dogs initially developing soft tissue swelling and a radiolucent line parallel to the metaphysis. This progresses to areas of sclerosis, metaphyseal widening (also known as “flare”) and new bone formation (Grøndalen 1976, Demko & McLaughlin 2005). These radiographic changes were all seen within our population.

At the time of diagnosis, three dogs (7.7%) had neutrophilic joint inflammation on synovial fluid analysis. From the literature, synoviocentesis does not appear to be routinely performed on dogs with metaphyseal osteopathy. There are two cases where this has been reported previously. A British Shorthair with metaphyseal osteopathy was reported to have neutrophilic joint fluid (Adagra *et al*. 2014). The other case was a dog that had normal synovial fluid with no evidence of inflammation (Greenwell *et al*. 2014). From the few previous reports and few cases in our population, it is not possible to determine whether the neutrophilic effusion was a reaction to the metaphyseal osteopathy diagnosed, or if there was concurrent immune‐mediated polyarthritis present.

This study provides insight into the disease progression, relapse episodes and other conditions that affect dogs with metaphyseal osteopathy. Relapse in our population was defined as a recurrence of clinical signs associated with metaphyseal osteopathy before the dog reaching skeletal maturity. This was based on the definition of relapse used by previous authors (Franklin *et al*. 2008, Safra *et al*. 2013, 2016). Relapse rate was 32.0% (eight of 25); this was similar to other studies findings (15.2 to 50.0%) which also suggest that relapse episodes can occur until dogs reach skeletal maturity (Abeles *et al*. 1999, Safra *et al*. 2013). Length of time from diagnosis to relapse episode(s) has not been reported previously. In our population, the median time from diagnosis to relapse was 50 days (range, 7 to 212 days). A limitation to the diagnosis of relapse was that further investigations had not been performed for any of our cases during suspected relapse episodes. Ideally, radiography (and other investigations if warranted) would be carried out to confirm that metaphyseal osteopathy is present and to exclude any other disease processes with similar clinical signs (e.g. immune‐mediated polyarthritis). It is, therefore, possible that the relapse rate reported here, and in previous literature, may be in excess of the true relapse rate within the population.

Long‐term follow‐up in dogs with metaphyseal osteopathy has been described in this study. Interestingly, a proportion of our population suffered from concurrent, or future, suspected immune‐mediated conditions. Future conditions in our population were defined as those which occurred after dogs reached skeletal maturity. This is in keeping with previous reports that relapse episodes of metaphyseal osteopathy can occur up to the point that dogs are skeletally mature (Abeles *et al*. 1999, Safra *et al*. 2013). In this study, there were four of 25 (16.0%) dogs that developed future immune‐mediated diseases. Aside from a case report of concomitant juvenile cellulitis in an Australian shepherd puppy (Wentzell 2011), the incidence of immune‐mediated conditions in dogs with metaphyseal osteopathy has not been reported. To the authors' knowledge there is no current literature reporting the incidence of immune‐mediated diseases in the general population of dogs. An epidemiologic study of insured Nova Scotia duck tolling retrievers (NSDTR) revealed 0.44% suffered from an immunological disease (Bremner *et al*. 2015). Unfortunately, as they are a small select group of dogs, it is not possible to compare this group of NSDTR to either the general population of dogs or our study population. However, it may be suggestive that the rate of immunologic diseases in the general population of dogs is low. It remains unknown if the development of future immune‐mediated conditions is more common in dogs with metaphyseal osteopathy than the general population. However, given the large proportion of these conditions in our dogs, development of immune‐mediated disease should be taken into consideration.

Relapse rate was higher in the population of dogs that developed future conditions (60.0%) than the study population as a whole (32.0%). This may suggest that dogs that have relapse(s) of metaphyseal osteopathy could be more likely to develop a future immune‐mediated condition.

It has been proposed that metaphyseal osteopathy is immune‐mediated. Cytokine profiles in dogs show altered cytokine production during active disease and remission (Safra *et al*. 2013, 2016). This is suggestive that metaphyseal osteopathy may be an autoinflammatory bone condition similar to chronic recurrent osteomyelitis (CRMO) in children. This disorder is thought to develop due to an imbalance of pro‐inflammatory and anti‐inflammatory cytokines leading to an increase in osteoclast activation (Sharma & Ferguson 2013, Moghaddas & Masters 2015, Hoffman *et al*. 2017).

A large proportion of people with CRMO suffer from concurrent inflammatory conditions; 19% have skin manifestations (Girschick *et al*. 2018) and 10% have inflammatory bowel disease (Hoffman *et al*. 2017). Interestingly, people with an immune‐mediated disease have been found to be at greater risk of suffering another concurrent, or future, immune‐mediated condition (Robinson *et al*. 2006, El‐Gabalawy *et al*. 2010).

Chronic recurrent multi‐focal osteomyelitis (CRMO) is thought to be heritable, with several forms of the disease found to have a genetic molecular basis (Safra *et al*. 2016, Hoffman *et al*. 2017). Entire litters and closely related Weimaraners and Australian kelpie dogs have been affected by metaphyseal osteopathy, suggesting that it may be heritable (Woodard 1982, Abeles *et al*. 1999, Harrus *et al*. 2002, Safra *et al*. 2013, Greenwell *et al*. 2014). Within our study Border collies, Weimaraners and Labrador retrievers were common. It is difficult to comment on the significance of this in the absence of a control group of dogs without metaphyseal osteopathy during the same time period.

Limitations of our study include its retrospective nature, small sample size and lack of follow‐up in all cases. Treatment protocols were not standardised, and length of treatment was not recorded in many dogs. The follow‐up period varied between dogs which may have led to inaccuracies in reported outcome. It was also not possible to evaluate how all of the other conditions were diagnosed; therefore, the prevalence of immune‐mediated diseases may have been under‐ or overestimated. Given the retrospective nature of our study, it is difficult to draw conclusions about optimal treatment regime. Ideally, a prospective randomised controlled trial would be performed to evaluate the efficacy of NSAIDs *versus* prednisolone.

In summary, although short‐term outcome for metaphyseal osteopathy is excellent, with all dogs within the current study surviving to discharge from hospital, relapse of clinical signs appears common. Optimal treatment and the underlying aetiology remain unknown. The findings of this study suggest a large proportion of dogs diagnosed with metaphyseal osteopathy may go on to develop future immune‐mediated diseases, which should be considered in the long term.

### Author contributions

**Alison Linton Robertson:** Data curation (lead); formal analysis (lead); investigation (lead); methodology (lead); project administration (lead); resources (lead); writing – original draft (lead); writing – review and editing (lead). **Victoria Louise Black:** Formal analysis (equal); methodology (equal); supervision (equal); validation (equal). **Erika Villedieu:** Data curation (equal); validation (equal). **Katherine Elizabeth Clarke:** Data curation (equal); validation (equal). **Ian Faux:** Data curation (equal); validation (equal). **Alison Major:** Investigation (equal); validation (equal). **Sophie Adamantos:** Conceptualization (lead); formal analysis (equal); methodology (equal); supervision (equal); validation (equal).

### Conflict of interest

None of the authors of this article has a financial or personal relationship with other people or organisations that could inappropriately influence or bias the content of the paper.

## Supporting information


**Table S1**. Vaccination protocols received by dogs (information available for 27/36 vaccinated dogs).
