# Supplementary material for: Presenting signs and clinical outcome in dogs with metaphyseal osteopathy: 39 cases (2009–2018)
Source: J Small Anim Pract. 2022 Sep 19;64(1):35–42. doi: 10.1111/jsap.13554 (PMC10087536; doi:10.1111/jsap.13554)
Supplement: Supplementary file 1 — Table S1. Vaccination protocols received by dogs (information available for 27/36 vaccinated dogs). [file JSAP-64-35-s001.docx]

**Appendix**

| **Appendix 1. Vaccination protocols received by dogs (information available for 27/36 vaccinated dogs)** |  |
| --- | --- |
| **Vaccination type** | **Number of dogs** |
| DHPPi L2 | 10 |
| DHPPi L4 | 5 |
| DHPPi | 2 |
| DHP L2 | 4 |
| DHP L4 | 4 |
| DHPPi L2 and KC | 1 |
| DHP | 1 |
| Unknown - protocol not recorded | 9 |
| Total number vaccinated | 36 dogs |

DHPPi Nobivac^Ⓡ^ live attenuated vaccine for *distemper,* canine *adenovirus type 2, canine parvovirus and canine parainfluenza*

DHP Nobivac^Ⓡ^ live attenuated vaccine for *distemper,* canine *adenovirus type 2, canine parvovirus*

L2 Nobivac^Ⓡ^ inactivated vaccine for *Leptospirosis interrogans serogroup* canicola and icterohaemorrhagiae

L4 Nobivac^Ⓡ^ inactivated vaccine for *Leptospirosis interrogans* serogroup canicola serovar Portland-vere, serogroup Icterohaemorrhagiae serovar Copenhageni and serogroup Australis serovar Bratislava. *Leptospirosis kirschneri* serogroup Grippotyphosa serovar Dadas

KC Nobivac^Ⓡ^ live attenuated vaccine for *Bordetella bronchiseptica* bacteria and *canine parainfluenza* virus
